# “Who and how to screen for endogenous hypercortisolism among young women presenting with clinical hyperandrogenism and/or menstrual abnormalities ”

**DOI:** 10.1007/s40618-025-02537-0

**Published:** 2025-02-21

**Authors:** Francesco Ferraù, Ylenia Alessi, Federica Nista, Anna Roux, Diego Ferone, Emanuela Arvat

**Affiliations:** 1 Department of Human Pathology of Adulthood and Childhood “G. Barresi”, University of Messina, Messina, Italy; 2 Endocrinology Unit, “G. Martino” University Hospital, University of Messina, Messina, Italy; 3 Department of Biomedical, Dental, and Morphological and Functional Imaging Sciences, University of Messina, Messina, Italy; 4 Endocrinology Unit, Department of Internal Medicine & Medical Specialties, University of Genova, Genova, Italy; 5 Oncological Endocrinology Unit, Department of Medical Sciences, University of Turin, Turin, Italy; 6 Clinica Endocrinologica, IRCCS Ospedale Policlinico San Martino, Genova, Italy

**Keywords:** Cushing Syndrome, Women, Hypendrogenism, PCOS

## Abstract

Endogenous Cushing’s syndrome (CS) is rare, with an incidence of 0.7–2.4 per million population per year according to population-based studies. However, evaluation of patients presenting disorders potentially related to cortisol excess, and therefore with a ‘high risk of clinical suspicion’ profile, could bring out several unrecognized cases. CS represents one of the most challenging endocrine diseases, with clinical features overlapping with those of common conditions affecting general population, invariably resulting in potential mis- or delayed diagnosis with negative consequences in terms of morbidity and mortality. CS is remarkably prevalent among young females, variably presenting with menstrual irregularities and/or signs and symptoms of hyperandrogenism. Herein we briefly reviewed literature on prevalence and clinical impact of menses abnormalities, acne and hirsutism -also coexisting in the context of a polycystic ovary syndrome- in CS, aiming at clarifying if, when and how to screen for hypercortisolism young women with these disorders.

## Introduction

Endogenous Cushing’s syndrome (CS) is a rare but probably underestimated disease consequent to chronic cortisol excess, which cause several systemic comorbidities, severely affecting on quality and quantity of life [1, 2]. Avoiding delayed diagnosis, and ensuring a prompt treatment are crucial for positively impacting on morbidity and mortality [3, 4]. CS clinical picture is extremely variable, with some signs/symptoms very typical as purple striae or proximal myopathy, and others which are common in general population, such as obesity, diabetes, hypertension [5]. Likewise, menstrual abnormalities, acne, hirsutism and polycystic ovaries, that are frequently encountered in female patients with cortisol excess, are also rather common among young women. The low incidence of CS combined with the high prevalence of the above-mentioned clinical conditions in the general population, make the differential diagnosis very challenging. At-risk populations for CS have been suggested including patients with hypogonadotropic hypogonadism, young patients with hypertension, osteoporosis and vertebral or rib fractures, patients with central obesity and clinical signs for CS, and with adrenal incidentaloma [5–9]. According to Endocrine Society Guidelines, adult patients with unusual symptoms for their age (as osteoporosis, hypertension), with multiple and progressive symptoms, especially those more predictive of CS, and with adrenal adenoma should be screened [10]. However, the evidence about epidemiology of CS, diagnosis as well as clinical management in young women is very low. Currently, definite screening recommendations for CS in young female patients with menses disturbances, alterations of hypothalamic-pituitary adrenal axis, associated with clinical or biochemical hyperandrogenism, are not available. In this regard, a recent survey found that only 17% of endocrinologists and 6% of gynecologists screened patients with these clinical features for CS, suggesting uncertainty in screening strategy and differential diagnostic work-up in these peculiar clinical contexts [11].

Herein, we briefly reviewed literature on prevalence and clinical impact of menses abnormalities, acne and hirsutism -also coexisting in the context of a polycystic ovary syndrome (PCOS)- in CS, aiming at clarifying if, when and how to screen young women with these disorders for hypercortisolism.

### Menstrual abnormalities, clinical hyperandrogenism, PCOS-like features and endogenous Cushing’s syndrome

Menstrual abnormalities and gonadal dysfunction are common in women with CS and, according to ERCUSYN registry data, could be early though non-specific manifestations of cortisol excess [4, 10, 12, 13]. The pathogenesis of menstrual disorders in CS is multifactorial, although still partially unclear. Cortisol excess negatively impact on gonadotropin levels with related estrogen deficiency, a picture consistent with secondary hypogonadotropic hypogonadism. The increased adrenal androgen levels and bioavailability, due to ACTH stimulation and to a lower hepatic production of SHBG respectively, impact on GnRH release [14]. Furthermore, the overweight of patients with CS could be associated with inappropriate synthesis of estrogens due to an increased aromatization of androgens, further contributing to acyclic feedback in the hypothalamic-pituitary-gonadal axis [14–16].

In a study involving 45 young women with Cushing’s disease (CD), 14 had oligomenorrhea (31.1%), 4 reported polymenorrhea (8.8%), and 15 experienced secondary amenorrhea (33%) [16]. Interestingly, these authors observed that patients complaining about amenorrhea had higher mean cortisol levels and lower either estradiol or SHBG levels. Considering the most recent data from the European Registry on CS, including 1225 women, menstrual disorders have been identified in more than half of patients (about 60%) [17]. Women with CD presented menstrual irregularities more frequently than those with adrenal CS (64% vs. 45%; *p* < 0.01). However, it is known that patients with CD usually show excess of both cortisol and androgens, while in adrenal CS androgen release could be inhibited [18]. Menstrual abnormalities were also observed in 43% of patients with ectopic CS. Moreover, in a recent study, among CS women aged < 45 years, at baseline evaluation, 43% had oligomenorrhea which was the main reason to start the diagnostic work-up for CS in half of those with CD and in 25% with adrenal CS [12].

In CS patients, the increase in androgen levels and the estrogen deficiency -and therefore their imbalance- as well as other direct and indirect cortisol excess-related effects on skin, pilosebaceous unit and hair follicles, are mainly responsible for the increased prevalence of acne and hirsutism.

Hirsutism may be the result of either androgen excess or increased sensitivity of the hair follicles to normal levels of androgens [19]. In general, up to 80–90% of cases of hirsutism, depending on age, race and ethnicity, show an underlying androgen excess disorder. Among this group, the most common disorder is PCOS (70–80% of cases), followed by idiopathic hyperandrogenism (15%), idiopathic hirsutism (10%), non-classic congenital adrenal hyperplasia (3%), hyperandrogenism with insulin resistance and acanthosis nigricans (3%), ovarian or adrenal androgen-secreting tumors (0,3%) and iatrogenic hirsutism, mainly due to the use of androgens, anabolic steroids and valproate. However, hirsutism in CS, as in other endocrine diseases such as hyperprolactinemia, acromegaly or thyroid disorders, is uncommon as isolated manifestation since is generally associated with other specific features of the underlying disease [20, 21]. The Endocrine Society guidelines report a prevalence of hirsutism of 48% in CS [10], while in the ERCUSYN European Registry hirsutism was found in 93% of women with ectopic ACTH CS and its prevalence was significantly higher compared with that in the other groups of CS (63% in the CD and 37% in patients with adrenal CS) [17]. Furthermore, Broder et al. found that hirsutism is at least 5 times greater in patients with CS compared to patients without CS and that the relative risk increases even more when hirsutism is associated with hypertension, type 2 diabetes mellitus, fatigue, psychiatric disorders or serious infections [22].

Acne may also be a clinical consequence of CS-related hyperandrogenism. The etiopathogenesis of acne is complex and not yet fully elucidated. It appears to involve an interaction among genetic predisposition, hormonal factors, such as hyperandrogenism, and chronic activation of the innate immune system overlapping with external factors, such as daily stress, diet, use of tobacco and cosmetics [23, 24]. In a study focused on CS population, the prevalence of acne and hirsutism was 56% [25]. Moreover, Erden et al. showed a major prevalence of acne in patients with exogenous CS, while hirsutism seemed to be more frequent in patients affected by endogenous CS [26].

On the other hand, menstrual abnormalities and clinical hyperandrogenism (acne, hirsutism) are features of PCOS, which in turn is commonly seen in CS patients, with substantial overlap between these conditions. Indeed, polycystic ovaries, oligo-anovulation, oligo-amenorrhea, hirsutism, acne, overweight/obesity and other glucometabolic abnormalities, including type 2 diabetes and insulin resistance, may be variably present in PCOS and CS patients. As shown by a prospective study, menstrual irregularity is seen in 70–80% of the female population with CS, with 46% of these patients showing features of PCOS [27, 28]. Overlapping between PCOS and CS is even more evident among those patients with moderately high glucocorticoid levels which generally do not lead to hypogonadotropic hypogonadism as in florid CS [29]. However, even in this latter condition PCOS phenotype could be due to low SHBG, increased androgens and to the effect of hyperinsulinemia on ovaries [18, 27]. In this context, Brzana et al. retrospectively reviewed records of female patients of reproductive age with CD and verified how many had been previously mis-diagnosed as having only PCOS. Among 50 patients with pathologically proven CD half had previously been diagnosed with and treated initially for PCOS. Hirsutism and menstrual abnormalities were more common in the group with an initial PCOS diagnosis than in the group with an initial CD diagnosis [30].

However, while it is undoubtful that oligo-amenorrhea and clinical hyperandrogenism are frequent in CS female patients, it is undeniable that these disorders have considerably different prevalence in general population. Indeed, whereas CS is a rare condition, menstrual abnormalities are frequently encountered in young women [10, 31], hirsutism affects 5 to 15% of women, acne has been reported to affect not only adolescents, but also an increasing number of adult women older than 25 year old, with a prevalence estimated between 12 and 54% [22], while PCOS is diagnosed in about 5–20% of women of reproductive age, representing the most common endocrine problem in this patient population [28, 32]. Therefore, identification of women with CS within large cohorts of patients presenting with irregular menses and/or androgen excess disorders represents a diagnostic challenge for the endocrinologist. On the other hand, a correct diagnosis is crucial since endogenous cortisol excess has multiple deleterious systemic effects but is curable, while oral estrogens often used to treat hyperandrogenism may be detrimental in CS patients because of their high thrombotic risk.

### WHO to screen for endogenous Cushing’s syndrome among patients with menstrual abnormalities and/or clinical hyperandrogenism?

Taken for granted that mass screening of patients with menses alterations, hyperandrogenism and PCOS-like features is not feasible, other clinical features, indicating which women should be screened for cortisol excess must be detected.

In detail, as for all the neuroendocrine causes of secondary amenorrhea, it has been proposed to carefully consider screening for endogenous hypercortisolism in women with menstrual abnormalities, especially if there are other signs and/or symptoms at the clinical examination that could suggest CS (e.g., skin alterations, proximal muscle atrophy, osteoporosis/bone fractures, hypertension, and glucose imbalance) [13, 33].

In patients presenting with hirsutism and acne, a detailed history and a focused physical examination are essential in order to determine the onset and rate of progression of the disorders, the use of interfering drugs, the family history and the presence of other signs and symptoms of hyperandrogenism (menstrual irregularities, alopecia, acanthosis nigricans and virilization) or signs and symptoms of other endocrine disorders that might be associated with hirsutism [11, 19]. In particular, the association of acne and hirsutism with central obesity, facial fullness, plethora, proximal muscle weakness, purple skin striae, thin skin, easy-bruising, buffalo hump and supraclavicular fat may suggest the diagnosis of CS [20]. Accordingly, the Endocrine Society Clinical Practice Guideline for Evaluation and Treatment of Hirsutism in Premenopausal Women recommend assessing for CS in female presenting with hirsutism only if other features of this condition are present [34].

Atrophic and purple striae, proximal myopathy, easy bruising, and thin skin are more frequently encountered in CS than in PCOS [11, 32]. Moreover, hypercortisolism leads to impaired BMD and increased risk of fractures, while women with PCOS generally have a preserved BMD [29]. A case-control study on the relative risk (RR) of individual and combined conditions among patients with CD versus matched non-CD controls, showed how RR of any one of 10 key conditions selected by expert opinion (localized adiposity, hirsutism, facial plethora, polycystic ovary syndrome, abnormal weight gain, hypokalemia, deep venous thrombosis, muscle weakness, female balding, osteoporosis) was ≥ 5 times greater in CD compared to non-CD, and nearly all dyads had RR ≥ 5 [22]. Therefore, where clinical signs of PCOS overlap to a greater extent with cortisol excess-related typical features, rates of detection of CS are much higher.

Finally, hypercortisolism should be evaluated in all patients with menstrual abnormalities, hirsutism, acne, or suspected PCOS, in presence of clinical signs suggestive of CS, bearing in mind the intertwined vicious circle connecting these conditions.

### HOW to screen patients with menstrual abnormalities and/or clinical hyperandrogenism for endogenous Cushing’s syndrome?

The screening evaluation for CS is reported in Endocrine Society’s Clinical Practice Guidelines, which recommended tests to assess the hypothalamic–pituitary–adrenal (HPA) axis integrity, including, as first line evalutation, 24-h urine free cortisol (UFC) excretion, late-night salivary cortisol (LNSC) and 1-mg overnight dexamethasone suppression test (1-mg DST), according to the suitability of each test to a particular clinical setting [10]. The sensitivity of 1-mg DST ranges from 91 to 97%, while specificity ranges between 87 and 94% based on different studies [35]. For 24-h UFC, the sensitivity and the specificity ranged from 90 to 98% and from 84 to 97%, respectively, while LNSC showed a sensitivity ranging from 83 to 100% and a specificity ranging from 84 to 97% [35].

The screening tools used to evaluate CS in women affected by menstrual irregularities, hyperandrogenism and/or PCOS features, as well as the prevalence of CS in these women, are reported in Table 1.

**Table 1:** Screening tests for endogenous hypercortisolism among young women presenting with clinical hyperandrogenism and/or menstrual abnormalities

Test	Reference	Patients (*n*.)	Menopausal status	Inclusion criteria	Test only if suggestive clinic	Cut-off (if reported)	Prevalence
**24 h-UFC**	Vexiau et al., 1990 [43]	87	Pre-menopausal	Acne and/or hirsutism	No	Not specified	0 (0%)
	O’Driscoll et al., 1994 [44]	350	Pre-menopausal (n.322) post-menopausal (n.28)	Skin manifestation of androgen excess (androgenic alopecia and/or hirsutism)	Yes	Not specified	0 (0%)
**1 mg DST**	Barbieri et al., 1990 [45]	100	Not specified	Hyperandrogenism (hirsutism, fertility problems or menstrual irregularities)	Yes	Not specified	1 (1%)
	Zargar et al., 2002 [46]	142	Pre-post menopausal	Hirsutism	Yes	Not specified	0 (0%)
**4 mg DST**	Unluhizarci et al., 2004 [47]	168	Pre-menopausal	Hirsutism	Yes	< 3 mcg/dl	1 (0.6%)
	Karaca et al., 2013 [48]	105	Pre-menopausal	Hirsutism (FG score ≥ 8)	No	< 1.8 mcg/dl	0 (0%)
**24 h-UFC** ** + 1 mg DST + LNSC**	Braun et al., 2022 [49]	28	Pre-postmenopausal	“PCOS” symptoms (acne, hirsutism and/or menstrual changes)	No	24 h-UFC (< 150 mcg/24 h) 1-mg DST (< 2.0 mcg/dL) LNSC (< 1.5 ng/mL)	6 (21.4%)
**24 h-UFC or 1 mg DST**	Corenblum et al., 1994 [50]	88	Pre-menopausal	Hirsutism (FG > 8), oligomenorrhea and/or obesity (BMI > 25 kg/m2)	No	Not specified	15 (17%)
	Azziz et al., 2004 [51]	873	Pre-post menopausal	Oligo-/amenorrhea, ovulatory dysfunction, excess hair growth, virilization, alopecia or acne	yes	Not specified	0 (0%)
	Glintborg et al., 2004 [52]	340	Pre-menopausal	Hirsutism	yes	Not specified	1 (0.3%)

UFC: urinary free cortisol; DST: dexamethasone; LNSC: late night salivary cortisol

The most used tests were 1-mg DST and 24-h UFC, but none of the studies reported their sensitivity and the specificity in these patient cohorts; moreover, the screening tests were mostly performed only in presence of a suggestive phenotype. Overall, most of the studies reported that hypercortisolism is rarely detected among premenopausal women with clinical hyperandrogenism, menstrual abnormalities and/or PCOS-like phenotype. Indeed, it is worth to be mentioned that no prospective study has so far been designed to evaluate the prevalence of CS among patients with PCOS.

In agreement with the evidence that none of the screening test has proven fully capable of identifying all cases of CS and all provided false-positive results in patients with non-neoplastic hypercortisolism [36, 37], PCOS women have been found with mildly increased UFC (25–50% of patients), apparent lack of cortisol suppression after DST (5–15% of patients) and increased midnight serum cortisol (MNC) concentrations (20% of patients), regardless of BMI [18, 28, 38]. Nevertheless, the 1-mg DST has shown a high diagnostic accuracy also in this screening setting.

Moreover, in a retrospective study carried on obese women (OB) of reproductive age (including PCOS patients) who were screened for CS and compared with premenopausal obese women with active CS, DST showed the greatest specificity and diagnostic accuracy in differentiating CS from PCOS and OB (according to the 138 nmol/l criterion) while MNC and UFC displayed a similar discriminatory value. However, by using a lower threshold (50 nmol/l) as response criterion, there were no diagnostic differences between DST and the other tests. Specificity and diagnostic accuracy of UFC measurement was lower in PCOS than in OB, while there were no differences between groups for DST and MNC. These results indicate that the 1-mg DST and MNC are unaffected by the presence of PCOS and can be safely used to screen for CS premenopausal obese women with PCOS, while caution should be exercised in interpreting mildly elevated UFC levels in these patients [39].

It has to be outlined that in these patients a complete medication history should be collected, since serum cortisol levels can be increased by oral contraceptives, as well as by spironolactone, drugs often taken by female patients with menstrual abnormalities or hirsutism. Indeed, estrogen treatment can increase cortisol-binding globulin (CBG) and lead to a false positive result on DST. Patients who have already started estrogen therapy before the screening for CS should stop the treatment for a period of about 6 weeks before planning a DST. When withdraw of oral estrogen is unfeasible, proper UFC assessment is recommended [10]. Alternatively, LNSC, that measures the free fraction of cortisol, and therefore is not influenced by estrogen treatment, is a valid option. In this regard, Issa et al. have recently demonstrated a strong correlation between salivary cortisone and serum cortisol after 1-mg DST [40]; moreover, LNSC levels in patients with CS have been found significantly higher than those reported in obese and non-obese women with PCOS [41]. LNSC has also the advantage of being suitable for outpatients, given the non-invasive, stress-free and easy collection of saliva [35, 38, 42] However, as circadian rhythm is altered in many patients with depressive illness and in shift workers, causing false positive results, these conditions must be carefully evaluated and at least two LNSC measurements in consecutive days are needed.

## Conclusions

CS is a challenging diagnosis, often delayed because of the large overlap of clinical picture consequent to cortisol excess with conditions or disorders with high prevalence in general population. In this setting, identifying at-risk population with features suggestive of cortisol excess would lead to define proper screening strategy reducing diagnostic delay and consequently disease burden. Clinical hyperandrogenism (acne, hirsutism) and/or menstrual irregularity, often configuring PCOS, are frequent finding in young women with CS. At the same time, PCOS-like features are a recurrent referral reason for endocrinological evaluation and show a not negligible prevalence in general population. According to literature data, prevalence of CS in unselected patients with clinical hyperandrogenism/menses abnormalities and PCOS-like features is very low and would not justify a screening for HPA axis disorders, except for young women showing other typical signs/symptoms of cortisol excess. In case of high clinical suspicion, diagnostic work-up can rely on the use of 1 mg-DST and LNSC which have shown to adequately perform in this clinical setting.
